# Low-Level Fluvalinate Treatment in the Larval Stage Induces Impaired Olfactory Associative Behavior of Honey Bee Workers in the Field

**DOI:** 10.3390/insects13030273

**Published:** 2022-03-10

**Authors:** Chong-Yu Ko, Yu-Shin Nai, Wei Lo, Chun-Ting Chen, Yue-Wen Chen

**Affiliations:** 1 Department of Biotechnology and Animal Science, National Ilan University, Yilan City 260, Taiwan; ricardo7677@gmail.com (C.-Y.K.); w8221902@hotmail.com (W.L.); beecct@yahoo.com.tw (C.-T.C.); 2 Department of Entomology, National Chung Hsing University, Taichung City 402, Taiwan; ysnai@nchu.edu.tw

**Keywords:** fluvalinate, honey bee, sublethal dosage, proboscis extension reflex

## Abstract

**Simple Summary:**

There may be high amounts of fluvalinate residue in bee colonies and products. It is usually used to control the varroa parasite on bees, but it may lead to adverse effects on both larvae and adults. In this study, we found that feeding with fluvalinate at a dose of 40 ng/larva could result in declined brood-capping, pupation, and eclosion rates. In addition, the olfactory associative behavior of adult bees was impaired after they were treated with a sublethal dose of 0.004 ng/larva in the larval stage. These findings suggest that a sublethal dose of fluvalinate given to larvae has a huge negative effect on adult honey bee workers, affecting their subsequent associative ability. It may further lead the entire colony to a detrimental developmental trend.

**Abstract:**

Fluvalinate is a widely used insecticide for varroa mite control in apiculture. While most beekeepers have ignored the effects of low levels of fluvalinate on bees, this study aims to demonstrate its effects at very low concentrations. We first used fluvalinate doses ranging from 0.4 to 400 ng/larva to monitor the capping, pupation, and emergence rates of larval bees. Second, we used the honey bees’ proboscis extension reflex reaction to test the learning ability of adult bees that were exposed to fluvalinate doses from 0.004 to 4 ng/larva in the larval stage. The brood-capped rate of larvae decreased dramatically when the dose was increased to 40 ng/larva. Although no significant effect was observed on brood-capping, pupation, and eclosion rates with a dose of 4 ng/larva, we found that the olfactory associative behavior of adult bees was impaired when they were treated with sublethal doses from 0.004 to 4 ng/larva in the larval stage. These findings suggest that a sublethal dose of fluvalinate given to larvae affects the subsequent associative ability of adult honey bee workers. Thus, a very low dose may affect the survival conditions of the entire colony.

## 1. Introduction

Exposure of honey bees (*Apis mellifera*) to various environmental pesticides when they are collecting nectar and pollen represents a serious problem [1,2,3]. Honey bees can be affected by contaminated nectar and pollen that are brought back to the hive from foraging on flowers that have been sprayed with pesticides. Moreover, they are also affected by applications of medicine aimed at controlling the widespread honey bee parasite varroa mite (*Varroa destructor*), which has been closely linked to colony losses and the mortality of honey bees [4,5,6]. Some medicines or pesticides are applied inside bee colonies by the beekeepers themselves. Among these in-hive pesticides, fluvalinate is one the agents used worldwide to control *V. destructor* in honey bee colonies [7].

Fluvalinate belongs to the pyrethroid family of neurotoxins, which prolong membrane depolarization by targeting voltage-gated sodium channels [8,9,10]. Pyrethroids decrease the kinetics of channel closure and block channels in their existing state. Through its mode of action, fluvalinate mainly results in hyperexcitability and can lead to musculature changes that culminate in paralysis or death. However, after several decades of intensive and repetitive use of fluvalinate, a reduction in its efficacy and the impact of selective pressure may favor the emergence of resistant mite populations [11,12,13,14,15,16], since it has been thought to have low toxicity in bees [17]. Currently, some investigations have reported fluvalinate-resistant mites [18,19,20,21,22,23]. The potential routes of exposure and corresponding responses of honey bee larvae have not been thoroughly studied [24].

The hive is a shelter for bees, and fluvalinate is used as a medicine to control varroa mites by applying it to the bee colony. Honey bees come in direct contact with fluvalinate during in-hive applications. Adult honey bees pass over the commercial fluvalinate strips used by beekeepers, then spread the chemical throughout the hive and subsequently to the mites. Newly emerged generations may also be exposed through contact with fluvalinate residue in honey and bee wax [25,26]. Treatment with fluvalinate can have negative effects on the short-term learning, long-term responsiveness to sucrose, and survival of adult honey bees [27]. Moreover, fluvalinate can be transmitted to larvae through bee wax [24] and food by trophallaxis in the colony after sub-lethal exposure [28]. Few studies have investigated the effects of fluvalinate on adult bee cognition [27,29,30,31] and the effects of sublethal doses of fluvalinate on bee larvae [24,32,33,34]. Therefore, we evaluated the effects of exposing larvae to fluvalinate contamination in the field by feeding purified fluvalinate to honey bee larvae during their development and examined the learning ability of adult bees that emerged from fluvalinate-polluted larvae.

## 2. Materials and Methods

### 2.1. Experimental Honey Bees

Healthy honey bees (*Apis mellifera*) were selected from the NIU experimental apiary colonies (National Ilan University, Taiwan; GPS coordinates: N24.747278, E121.746200). There were 7 combs in each experimental colony, containing over 20,000 bees and a healthy laying queen. Oxalic acid was the only reagent used to control varroa mites in the experimental apiary. The tested colonies did not experience contact with fluvalinate before treatment and they were without varroa mite control for 8 weeks. Before starting the experiments, all colonies were checked to make sure they were not parasitized by the varroa mite by washing them with water [35]. The colonies underwent normal management weekly without mobilization.

### 2.2. Preparation of the Fluvalinate Solution

#### 2.2.1. Purification of Fluvalinate

Since the price of fluvalinate standard powder is USD 81.40 for 100 mg (purity = 96.3%), we prepared purified fluvalinate as a stock solution for subsequent experiments. In order to obtain the amount of fluvalinate to meet the needs of the field experiment, the powder was purified from the commercial fluvalinate pesticide (24% *w*/*w* emulsion, Makhteshim Chemical Works Ltd., Beer Sheva, Israel). Briefly, 5 g of commercial fluvalinate was diluted 10-fold in double-distilled H_2_O (ddH_2_O) and mixed well with 60 g silica gel (Silicycle R10040B, Midland Scientific Inc., La Vista, NE, USA). Next, the mixed fluvalinate–silica gel was transferred into a burette (24/40 2.5 mm) and eluted with 500 mL of n-hexane (J.T. Baker^®^, Inc 222 Red School Ln., Phillipsburg, NJ, USA). The eluted solvent was collected and the purification procedure was repeated twice. The total eluted solvent was collected, then evaporated by using a rotary evaporator system (Buchi Rotavapor R-215, BUCHI Taiwan Ltd. Taipei, Taiwan) under reduced pressure to obtain purified fluvalinate. The purified product was yellowish in color. One gram of purified fluvalinate was dissolved in 1 mL of dimethyl sulfoxide (DMSO, purity 99.9%, Merck Ltd. Taiwan, Taipei, Taiwan) and prepared as a stock solution at a concentration of 1 g/L in ddH_2_O for the consequent experiments and high-performance liquid chromatography (HPLC) assay. 

#### 2.2.2. High-Performance Liquid Chromatography (HPLC) Assay

Fluvalinate standard (Chem Service, https://www.chemservice.com/tau-fluvalinate-n-13263-100mg.html (accessed on 8 March 2022) and purified fluvalinate stock solution (1 g/L) were subjected to HPLC analysis by an Agilent 1200 Series chromatograph (Agilent, Santa Clara, CA, USA). Sample separations were performed on an Agilent ZORBAX SB-C 18 (Agilent, Santa Clara, CA, USA)5 μm, 4.6 mm × 250 mm reversed-phase analytical column. For chromatographic conditions, the elution was carried out with a fixed ratio mixture consisting of 2 solvents (A + B = 70 + 30, *v*/*v*). Solvent A was 100% acetonitrile and solvent B was 2% acetic acid in acetonitrile. The flow rate was 1.5 mL/min, the column was kept at room temperature, and detection was performed at a wavelength of 230 nm. The injection volume consisted of 10 μL. HPLC patterns of the fluvalinate standard and purified fluvalinate stock solution were compared at a concentration of 1 g/L. HPLC results indicated that the patterns of purified fluvalinate stock solution and fluvalinate standard were identical (Figure S1), suggesting that the purification method was workable and efficient (purity > 90%).

#### 2.2.3. Preparation of Working Fluvalinate Solution

Fluvalinate stock solution (1 g/L) was serially diluted to final concentrations of 0, 0.1, 1, 10, and 100 mg/L in 0.1% DMSO for the following experiment; 0.1% DMSO in water was prepared as the negative control (0 g/L).

### 2.3. Fluvalinate Feeding and Adult Bee Collection

Feeding and recording were performed according to our previous report [36]. Each treatment included 4 replicates (4 colonies) and 50 cells containing 1-day-old worker larvae in a comb, which were randomly selected and marked. For every dose, 1 μL of fluvalinate solution was pipetted per cell once a day from day 1 to day 4. Control larvae (0 mg/mL) received the same amount of 0.1% DMSO. This resulted in total fluvalinate concentrations of 0.4, 4, 40, and 400 ng per larva for the 4 treatment doses. After adding fluvalinate, the treated combs were returned to their original hives. The mortality and capping rates of marked cells were assessed on day 7. On day 15, the pupae in the marked areas were taken out from capped cells and put into 24-well tissue culture plates with double-layer tissue papers (Kimwipes, Kimberly-Clark Corporation, Irving, TX, USA) at the bottom of each well. The plates were kept in an incubator at 34 °C and 70% relative humidity until emergence to record the eclosion rate. The adult honey bees were collected and subjected to the following experiment.

Capping rate = (count of capped brood cells/number of marked cells) × 100%

Pupation rate = (count of pupae/number of marked cells) × 100%

Eclosion rate = (count of alive honey bees/number of marked cells) × 100%

### 2.4. Proboscis Extension Reflex (PER) Test

To investigate the effect of a sublethal dosage of fluvalinate on honey bees, their larvae were treated as described above but with different concentrations of fluvalinate, from 0.0001 to 1 mg/L, equivalent to a total of 0.0004 to 4 ng of fluvalinate received by each larva. Newly emerged honey bees were labeled with different colors on the scutum of the mid-thorax and then put back into a normal colony to experience the same socialization. After 15 days, the honey bees were collected into a plastic box until processing for the experiment. The numbers of adult bees collected in each experimental group were as follows: 30 in the blank unfed group, 30 in the 0.1% DMSO group, 37 in the 4 ng/larva group, 37 in the 0.4 ng/larva group, 25 in the 0.04 ng/larva group, 26 in the 0.004 ng/larva group, and 30 in the 0.0004 ng/larva group. Conventional conditioning of the PER was performed to test the olfactory associative behavior of fluvalinate-treated honey bees. Honey bees were placed in a dark incubator at 25 °C and 40% relative humidity, where they were starved for 4 h before the test. At the third hour of the starving period, the honey bees were anesthetized by being placed in a 4 °C ice bucket for 5 to 10 min. After anesthesia, they were fixed onto 1000 μL pipette tips by a beeswax/resin mixture and left for 1 h until their physiological condition recovered. Cotton swabs dipped in 50% (*w*/*v*) sucrose solution were applied to the honey bees’ antennae. Honey bees with a normal PER were tested for their olfactory associative behavior, whereas those that could not produce a PER response were eliminated. Approximately 30 honey bees per group were tested for their olfactory associative behavior after various treatments. The PER training method of Yang et al. [37] was used. The test for PER using odorous stimulation was only conducted once the honey bees associated this stimulation with sugar water. Each honey bee went through 4 associative tests (conditioning 1–4) and the tests were held 20 min apart.

PER response rate = (count of honey bees with response/number of tested honey bees) × 100%

### 2.5. Statistics

The effects of fluvalinate on the larval capping, pupation, and eclosion rates were analyzed by using Tukey’s honestly significant difference test (*p* < 0.05). The honey bees’ olfactory associative behavior 15 days after emergence was analyzed by the two-tailed Kruskal–Wallis H test (*p* < 0.001) and two-tailed Mann–Whitney U test with Dunn–Šidák correction at the 95% confidence level as a post hoc test. The data analysis was performed using SPSS (Version 20.0.0) to analyze statistical differences among treatments.

## 3. Results

### 3.1. Effects of Fluvalinate on Honey Bee Larvae Development

During this study, 0.1% DMSO, a chemical solvent, was used to confirm the non-effective influence of fluvalinate on honey bee development. According to our data, 0.1% DMSO had no significant effect on the brood-capping, pupation, and eclosion rates compared to the unfed control group (Table 1). The two higher doses (40 and 400 ng/larvae) caused high mortality during the larval stage. The brood capping rates were 3.33% for the 40 ng/larva group and 0% for the 400 ng/larva group (Table 1). The capping rates of these two groups were significantly lower than those for the other groups (*p* < 0.05). In the second high-dose group, even though the pupation rate was 3.33%, no pupa could enter the next instar for eclosion (Table 1). There were no eclosed adults at both higher doses; thus, the pupation and eclosion rates could not be measured (Table 1). For the two lower dosages (0.4 and 4 ng/larva), the brood-capping, pupation, and eclosion rates were not significantly different from either those of the 0.1% DMSO or unfed control group (Table 1). As shown in Table 1, the dose of 4 ng/larva was the lethal threshold. Thus, we further processed PER experiments for a dose of fluvalinate not exceeding 4 ng/larva.

### 3.2. Influence of Fluvalinate on Olfactory Associative Behavior of Honey Bees

**Figure 1:**
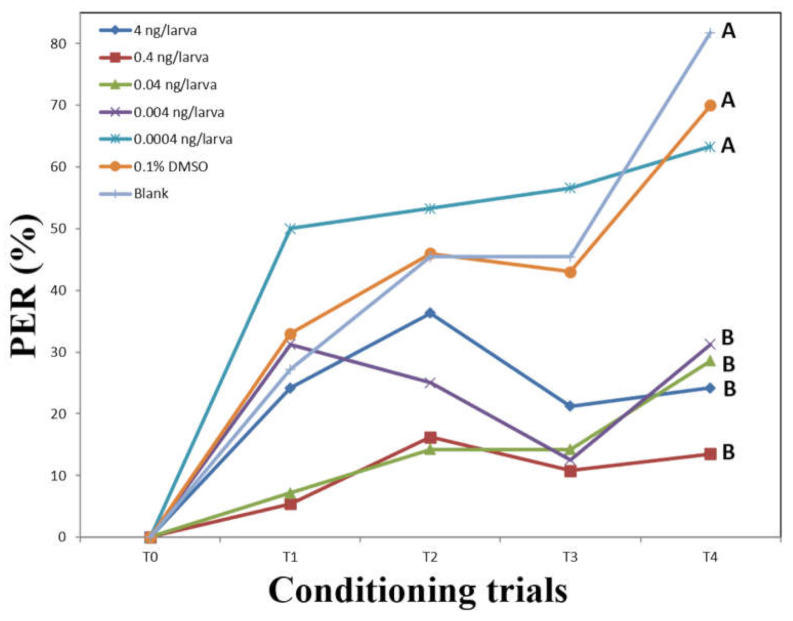
Olfactory associative behavior in honey bee adults following larval contamination with fluvalinate. T0 is trial to test normality of honey bees. T1, T2, T3, and T4 are conditioning trials. A and B indicate significant differences (*p *< 0.001) between treatments (two-tailed Kruskal–Wallis H test, *p *< 0.001, compared with two-tailed Mann–Whitney U test with Dunn–Šidák correction at a 95% confidence level).

The olfactory associative behavior of 15-day-old adult honey bees that emerged from larvae is shown in Figure 1. For the blank (unfed) treated group, the PER rate was 0% for conditioning trial 0 (T0), 27.2% for T1, 45.45% for T2, 45.45% for T3, and 81.8% for T4. For the 0.1% DMSO treated group, PER rates were 0, 33, 46, 43, and 70% for T0 to T4, respectively. The PER responses of these two groups showed a similar increased pattern of learning ability. Our results indicate that the influence of 0.1% DMSO on the olfactory associative behavior after eclosion can be ignored. The response rates of larvae treated with the lowest dose of 0.0004 ng fluvalinate were 0, 50, 53, 56.6, and 63.33% for T0 to T4, respectively (Figure 1). The rates for larvae treated with the dose of 0.004 ng fluvalinate were 0, 31.25, 25, 12.5, and 31.25% for T0 to T4, respectively (Figure 1). When the dose was increased to 0.04 ng, the response rates decreased to 0, 7.14, 14.2, 14.2, and 28.57% for T0 to T4, respectively. With the relatively high dose of 0.4 ng, the response rates further dropped to 0, 5.4, 16.2, 10.8, and 13.51% for T0 to T4, respectively. With the 4 ng fluvalinate treatment, the response rates were 0, 24.2, 36.3, 21.21, and 24.2% for T0 to T4, respectively (Figure 1). These results indicate that the PER rates of fluvalinate-treated groups were dose dependent. There were no significant differences in PER rates among the non-fluvalinate-treated groups (0.1% DMSO and blank) and the group treated with 0.0004 ng/larva of fluvalinate (*p* > 0.001) (Figure 1). The bees in the 0.1% DMSO, blank, and 0.0004 ng fluvalinate groups showed better olfactory associative ability than those treated with doses higher than 0.0004 ng/larva (*p* < 0.001). 

## 4. Discussion

### 4.1. Effects of Fluvalinate on Honey Bee Larval Development

The tested larvae were mostly raised by the colony, except when they were fed with fluvalinate by the experimenters. This direct feeding method is as close as possible to the natural conditions of a honey bee colony [37], in which the survival, capping, pupation, and eclosion rates of larvae can be affected by the hygienic behavior of nurse bees. The damage caused by nursing cannot be excluded when the nursing behavior is not measured. Our study showed no effect on larval development in the group receiving 0.1% DMSO (Table 1). Similar results were also reported when 1% or 0.1% DMSO was applied to honey bee larvae [37], demonstrating that 0.1% DMSO was not the affecting factor. Fluvalinate is commonly applied by beekeepers before the winter season to control the bee parasite *V. destructor*. Generally, fluvalinate can still be detected 30 days after application in the hive at an average (±SD) concentration of 1.29 ± 1.93 ng/bee [38]. This raises a concern that a sublethal dose existed in the bee colony for a long time, and the fluvalinate residue may exceed the doses used in this study. Benito-Murcia et al. [11] found the highest amount of fluvalinate residue in bee bread (1960.5 ± 139.2 ng/mL), which is the main source of nutrition for honey bee development. This might result in a cumulative effect of fluvalinate in the food storage system of honey bees. In our research, we found that 40 ng/larva of fluvalinate was a lethal dose to larval bees (Table 1). Compared to adults, larvae have up to 7-fold increased sensitivity to pesticides [34,39]. Since fluvalinate is more widely used in bee colonies and has been used for decades, these negative effects on bees should be noticed. In this study, we observed that the groups receiving doses lower than 4 ng/larva showed no significant decreases in the capping and pupation rates, motivating us to study other non-lethal effects of these doses.

### 4.2. Influence of Fluvalinate on the Olfactory Associative Behavior of Honey Bees

Since no significant lethal effect was observed on development with the 4 ng/larva treatment (Table 1), our data suggest that the olfactory associative behavior of adults was impaired by sublethal doses (4 to 0.004 ng/larva) of fluvalinate treatment in the larval stage (Figure 1). The Pavlovian conditioning method is widely used to assess honey bees’ ability to learn, wherein they are trained to respond to a sensory cue, typically an odor, paired with a nutritional reward [27,37]. Our understanding of the sublethal effect of fluvalinate on honey bee larvae is relatively poorer compared to what has been reported for adult honey bees. The olfactory associative ability results revealed that the groups receiving fluvalinate in doses over 0.004 ng/larva had a significantly lower ability than non-fluvalinate-treated groups (unfed and 0.1% DMSO) (Figure 1). 

Lin et al. [40] indicated that olfactory modulation in honey bees under sublethal exposure to fluvalinate is through the short neuropeptide F (sNPF) signaling pathways. Topical application of fluvalinate at sublethal doses is known to reduce the movement of individuals and their social exchanges with nest mates [41], which may lead to insufficient socialization, resulting in decreased learning capacity. However, the learning ability of the group treated with 0.0004 ng/larva of fluvalinate was not significantly different than the blank and unfed control groups. We found that there was dose-dependent effect in the groups treated with 0.004–0.4 ng/larva of fluvalinate, and the group with 0.4 ng/larva of fluvalinate had the worst PER response (Figure 1). 

Because the load of larval food before pupation is about 160 µL [42], it is possible that bee larvae are contaminated by environmental fluvalinate residue. We did not monitor the fluvalinate residue in the hive before the experiment. Fluvalinate could accumulate in colony products, especially in bee wax [11]. Although the pesticide had never been applied in these experimental colonies, it might have come from the foraging behavior of adult workers. Surprisingly, in the group treated with 4 ng/larva of fluvalinate, the level was a little higher than that in the groups with 0.4–0.004 ng/larva of fluvalinate groups, but it was not statistically significant. This is the first finding that has never been seen before, but we can still see a downward trend in the group’s PER response, which should predict a decreasing response if it continued for more trials. Compared to the result of Yang et al. [37], imidacloprid at a dose of 0.004 ng/larva did not cause impaired olfactory associative behavior, but in our study, only four trials of the PER experiment showed a dramatically decreased PER response under the same fluvalinate dose. This finding suggests that fluvalinate is a key influencing factor that is unfavorable to bees. The bee larvae would be exposed to high risk in their developmental environment, which could affect the subsequent associative ability of the adult bee workers. In this paper, we suggest that a very low dose of fluvalinate could also modulate the development of honey bee larvae, resulting in impaired PER.

## 5. Conclusions

The low level of fluvalinate application in honey bee colonies in the field may lead to adverse effects on both larvae and adults. The brood-capped rate of larvae was found to decrease dramatically when the treatment dose increased to at least 40 ng/larva of fluvalinate. Moreover, we found that the olfactory associative behavior of adult bees was impaired when they had been treated with fluvalinate at doses from 0.004 to 4 ng/larva in the larval stage. These results demonstrate that a sublethal dose of fluvalinate given to larvae affects the subsequent associative ability of adult honey bee workers. A sublethal dose of fluvalinate may indirectly affect the survival condition of the entire colony by reducing the learning behavior of fluvalinate-polluted workers. Thus, we suggest that beekeepers should reduce the use of fluvalinate. The efficacy of various organic materials in controlling varroa mites has been proven, thus mitigating undesired residues that may exert harmful effects on honey bees.

## Figures and Tables

**Table 1:** Effects of consecutive 4-day feeding of fluvalinate on 1-day-old larvae.

Fluvalinate (ng/Larva)	Larval Development		
	Capping Rate (%)	Pupation Rate (%)	Eclosion Rate (%)
400	0 ^b^	0 ^b^	0 ^b^
40	3.33 ± 6.7 ^b^	3.33 ± 6.7 ^b^	0 ^b^
4	92.3 ± 6.5 ^a^	88.5 ± 5.7 ^a^	80.00 ± 5.9 ^a^
0.4	95.7 ± 4.2 ^a^	86.7 ± 3.4 ^a^	82.68 ± 5.7 ^a^
0.1% DMSO	96.3 ± 4.3 ^a^	86.3 ± 8.6 ^a^	82.00 ± 8.7 ^a^
Unfed control	97.7 ± 2.9 ^a^	91.2 ± 3.9 ^a^	84.68 ± 5.7 ^a^

Once a day, 1 μL of fluvalinate per concentration was added to each larval cell from day 1 to day 4. Each assay contained 50 larvae in a colony and 4 replicates were tested. Mean ± SD are presented. Different letters in the same column indicate significant differences by Tukey’s honestly significant difference test (*p* < 0.05).

## Data Availability

The data presented in this study are available in article and Supplementary Material.

## Supplementary Materials

The following supporting information can be downloaded at: https://www.mdpi.com/article/10.3390/insects13030273/s1, Figure S1. Comparison of high performance liquid chromatography (HPLC) pattern of fluvalinate standard (A) and the purified fluvalinate stock (B) at the concentration of 1 g/L (in 0.1% DMSO).
