# Supplementary material for: Low-Level Fluvalinate Treatment in the Larval Stage Induces Impaired Olfactory Associative Behavior of Honey Bee Workers in the Field
Source: Insects. 2022 Mar 10;13(3):273. doi: 10.3390/insects13030273 (PMC8949447; doi:10.3390/insects13030273)
Supplement: Supplementary file 1 [file insects-13-00273-s001.zip › insects-1594438-supplementary.pdf]

(A) Fluvalinate standard

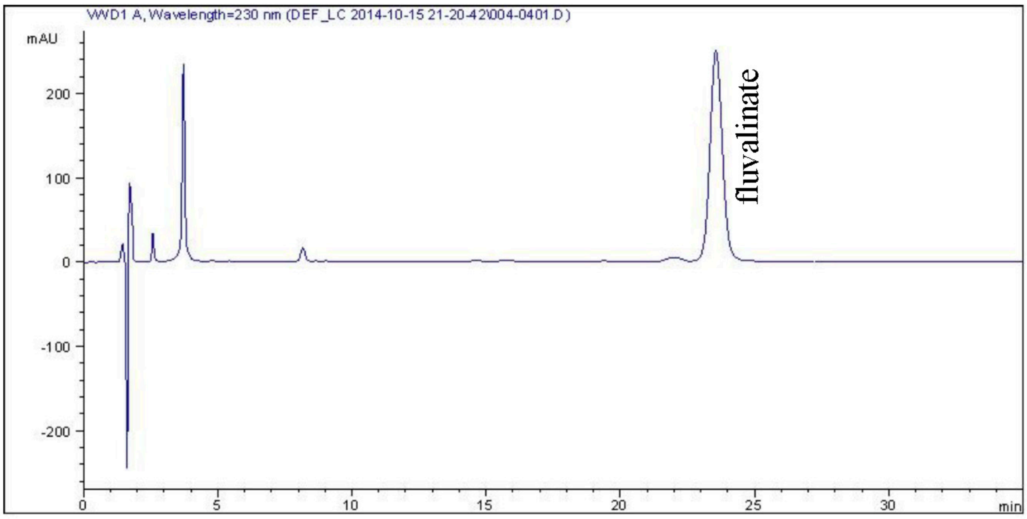

(B) Purified fluvalinate

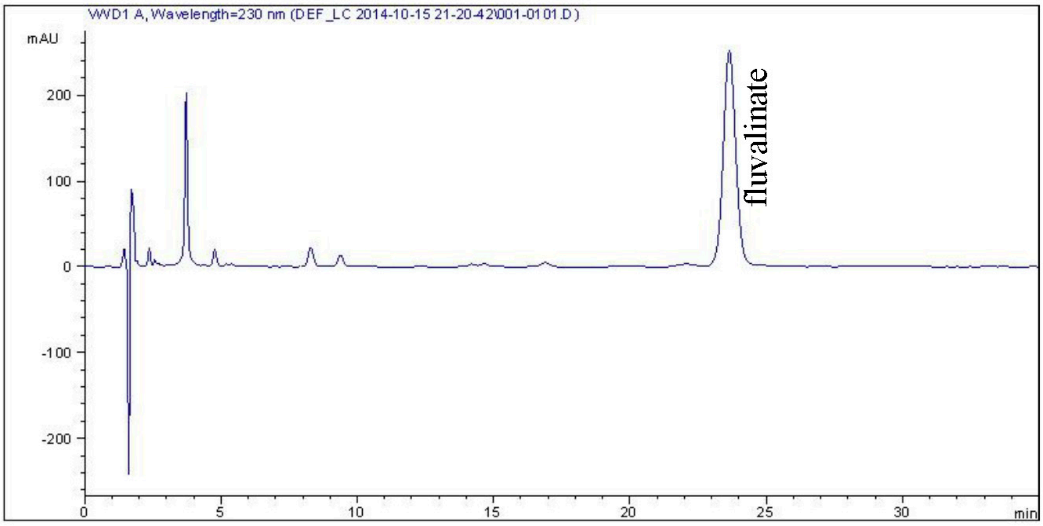

Figure S1. Comparison of high performance liquid chromatography (HPLC) pattern of fluvalinate standard (A) and the purified fluvalinate stock (B) at the concentration of 1 g/L (in 0.1% DMSO). The identical patterns were found in this assay.
